# Fish allergy-natural history and crossreactivity between fish species

**DOI:** 10.1186/2045-7022-1-S1-O26

**Published:** 2011-08-12

**Authors:** George Stavroulakis, Stavroula Giavi, Nikolaos Douladiris, Manolis Manousakis, Nikolaos G Papadopoulos

**Affiliations:** 1 2nd Pediatric Clinic, University of Athens, Allergy department, Athens, Greece

## Background

The clinical course of fish allergy is not sufficiently studied. Persistence seems to be the dominant pattern; nevertheless, a number of cases may overcome this allergy with time. Also, due to the high structural homology of parvalbumins from different fish species, crossreactivity among fishes is common.

## Objective

We are seeking to determine the clinical course of fish allergy in a Greek pediatric population and whether fish allergic children are able to tolerate some fish species without a reaction.

## Material and methods

105 children with a diagnosis of type I allergy to fish, based on history, specific IgE (sIgE, CAP) to codfish (f3) and skin prick tests (SPT) with commercial fish extracts, are included so far. All children undergo open food challenges (OFC) with canned tuna, fresh swordfish or fresh tuna in order to evaluate whether these fishes are tolerated or not by fish allergic children. Subsequently, those children showing signs of reduced clinical reactivity are subjected to double blind placebo controlled food challenges (DBPCFC) with codfish, followed by OFC to codfish in those with a negative DBPCFC, to evaluate the natural history.

## Results

a total of 71 challenges in 52 children were conducted so far. Forty one (91%) out of 45 children were negative to canned tuna- 3 were positive (2 anaphylactic reactions and 1 eczema flare up)- 1 OFC was inconclusive. Eleven (78%) out of 14 children were negative to fresh swordfish or fresh tuna- 2 were positive and 1 was inconclusive. Finally, 8 children underwent a DBPCFC to codfish and 4 were negative. OFC to codfish was also negative in those four children and they were considered fish tolerant. Both sIgE and SPT values showed a diminishing trend in these four patients. The mean time between first reaction and the achievement of tolerance for these four children was 9.25 years.

## Conclusion

The reduction of CAP levels and SPT diameter over time may indicate the diminishing sensitivity in fish allergic patients, most of which seem to be able to consume canned tuna, fresh tuna and fresh swordfish, although some reactors do exist even to those low allergenicity fishes.

